# Special Issue: Poultry Genetics, Breeding and Biotechnology

**DOI:** 10.3390/genes12111744

**Published:** 2021-10-29

**Authors:** Jun Heon Lee

**Affiliations:** 1 Division of Animal and Dairy Science, College of Agriculture and Life Sciences, Chungnam National University, Daejeon 34134, Korea; junheon@cnu.ac.kr; 2 Department of Bio-Big Data, Graduate School, Chungnam National University, Daejeon 34134, Korea; 3 Department of Bio-AI Convergence, Graduate School, Chungnam National University, Daejeon 34134, Korea

Poultry products, meat and eggs, are important sources of protein in the human diet worldwide. This means that there is a need for a persistent, sustainable, healthy, and adequate supply of poultry products on the market. Therefore, using accelerated production processes while conserving the nutritional benefits and minimizing the potential health hazards is required. Throughout history, with the domestication of poultry, there has been a tendency towards the selection and breeding of poultry species to maximize their production traits. These processes are ongoing. Despite the favorable traits of such developed breeds, there are some native poultry breeds being documented, signifying that further improvements are possible for traits such as disease resistance. In addition to the studies involved with traits related to meat quality and egg production, the major poultry, chicken, and duck species have been used in studies of developmental biology, oncogenesis, virology, and immunology.

With the first ever publication of the chicken genome in 2004, scientific studies in the field of “Poultry Genetics, Breeding, and Biotechnology” have improved tremendously. Nevertheless, the genetic backgrounds of various poultry characteristics and traits related to their economic importance still remain to be discovered. With the improvement of the novel genomic techniques, researchers have acquired a high potential for resolving the poultry species that directly influence their production or validate their genetic potential. Immense progress has been made in poultry genome-based research in recent years. For example, from 1957 to 2005, the growth rate of the broiler chicken has increased by more than 400%, and the feed conversion ratio (FCR) has decreased by 50% [1]. However, these changes have still not fulfilled human requirements. Therefore, further studies of poultry breeding, genetics, and biotechnology are required.

This Special Issue comprises 17 original research articles and two reviews that cover various aspects of genetics, breeding, and biotechnology with regard to poultry species.

Disease resistance in chicken is very important for the poultry industry, because it directly affects the production capacity and is also relevant to food security issues. A few research papers in this Special Issue cover this important theme, taking different approaches. With the goal of vaccine development, Võ et al. [2] investigated the microneme protein 2 (MIC2) and surface antigen 1 (SAG1) of *Eimeria tenella*, the genus responsible for the avian coccidiosis outbreaks. The etmic2 and etsag1 in Korean *E. tenella* isolates have been used for the analysis of genetic diversity and natural selection, while the information obtained is also important for designing effective coccidial vaccines. Another study describes the induction of host defense peptides (HDPs) in two chicken lines: one disease-resistant and one disease-susceptible line [3]. Based on the mRNA expression patterns of the HDPs, a higher expression was observed in the disease-resistant Fayoumi line than in the disease-susceptible Leghorn line, indicating that the HDPs are involved in disease resistance in chicken. Manjula et al. [4] investigated the chicken major histocompatibility complex (MHC)-B region using 11 MHC-linked microsatellite markers from four different countries (Sri Lanka, Bangladesh, South Korea, and Nigeria). Based on the Set 1 and Set 2 analysis, 409 haplotypes were identified. These haplotype data can be used further in the analysis of MHC polymorphisms for differential immune responses.

Muscle development is an important economic trait for broilers. In a study conducted by Zhang et al. [5] on the role of an miRNA and miR-21-5p, for the proliferation and differentiation of skeletal muscle satellite cells (SMSCs), the authors confirmed that KLF3 was the target gene of miR-21-5p. Therefore, this particular study demonstrated the function of miR-21-5p, which affects the growth and development of skeletal muscle in chickens.

With the publication of the chicken genome in 2004, large-scale genome research became popular. Consequently, in a study that was part of the Genome-Wide Association Study (GWAS) with a 60K single nucleotide polymorphism (SNP) chip, 12 significant SNPs for body weight at eight weeks of age were observed in Korean native chickens (KNCs). These can be used as selection markers for body weight [6]. Similarly, another study investigated the selection signatures between Korean native chicken and commercial chicken breeds using 600K SNP chip data [7]. Several regions for the selection signatures were identified, suggesting the use of these locations to identify the causative genes (and mutations) for improving meat- and egg-related traits in native chicken breeds.

Serotonin, 5-hydroxytryptamine (5-HT), is involved in many physiological functions in vertebrates. However, little information has been reported in avian species to date. Sun et al. [8] investigated the full-length cDNA and expression analysis of three serotonin receptor genes (HTR1B, HTR1E, and HTR1F) in chicken pituitaries. The results will improve our understanding of the serotonin function using the receptor genes in chicken.

The focus of Mukae et al. [9] has been directed toward transgenic chickens, with the aim of producing monoclonal antibodies (mAbs) in egg whites. Accordingly, genes for the heavy and light chains of humanized anti-HER2 mAb were inserted into the chicken ovalbumin gene locus using CRISPR/Cas9 methods. The results showed that chickens can be used efficiently for the production of mAbs and can even be used for commercial mAbs production.

Bone health is also becoming more important for both broilers and layers. Adhikari et al. [10] investigated the effect of 20(S)-hydroxycholesterol (20S) on mesenchymal stem cells isolated from the compact bones of broiler chickens (cBMSCs). The results suggested that 20(S) can have a significant function in the differentiation of chicken derived MSCs and can provide a number of clues regarding the regulation of skeletal, myogenic, and adipogenic differentiation in chicken. These results will also contribute to the maintenance of chicken bone health and meat quality. Jansen and colleagues [11] investigated the genes affecting bone breaking strength and bone mineral density in laying hens. The possible SNPs were identified using a machine learning algorithm called the random forests classification. From the results, 16 candidate genes were selected, and the SNP effects were estimated. The identified genes and SNPs could be used for decreasing bone disorders in laying hens.

The main roles of G protein-coupled receptors are regulating the functions of the central nervous system (CNS) and oocyte arrest in mammals. However, less information is available regarding their functions in birds. Li et al. [12] investigated three G protein-coupled receptors (GPR3, GPR6, and GPR12) and one novel GPR12-like receptor (GPR12L) and identified the structure, function, and expression of these receptors in chicken and duck. Moreover, the authors found that GPR12 expression was upregulated by progesterone. These data provide valuable information for readers, including the roles of these orphan receptors from a comparative endocrinology perspective in vertebrates.

Among poultry species, chicken is the most popular species in terms of its economic viability for farmers and food consumption. However, this Special Issue includes seven papers related to non-chicken poultry species, which have become more important as a food source worldwide. Three papers in this Special Issue present research results for geese, although on a population basis, duck is the second most popular poultry species in the world. Melak et al. [13] describe SNP markers for the marketing weight in male Yangzhou geese. The SNP markers were identified using the specific locus amplified fragment sequencing (SLAF-seq) method, with a bulked segregant analysis (BSA). Among the sequences with a 44.97 sequence depth, a total of 12,917 SNPs were identified. Finally, the authors identified 10 novel SNPs from the 31 most significant SNPs in relation to the target trait of marketing weight in geese. The expression analysis using quantitative PCR results indicated a significant difference in seven genes. These identified genes and SNPs will be useful markers for improving the marketing weight in geese. Another study of geese identified SNPs and conducted a phylogenetic analysis using genotyping-by-sequencing (GBS) for 12 conservative geese breeds, together with a commercial breed called White Kołuda^®^ from Poland [14]. Among the 3833 SNPs identified using the GBS method, 791 SNPs were finally selected for the population analysis. One of the identified variants was related to the EDAR gene, which affects plumage development and can be used for the selection of geese. Because the enzyme involved in goose follicular development is not currently well understood, Yuan et al. [15] investigated the expression of the SCD gene using quantitative reverse-transcription (qRT)-PCR. Furthermore, liquid chromatography-tandem mass spectrometry (LC-MS/MS) was used to investigate the function of SCD in granulosa cells (GCs). Based on the qRT-PCR and LC-MS/MS results, cholesterol and pantothenol (or pantothenate) were identified as potential metabolite biomarkers in relation to SCD-related lipid metabolism in goose GCs.

Papers have been published in this special edition concerning relatively less common species, which include turkey, Japanese quail, and pigeon, indicating the importance and potential of expanding poultry research. Bernini et al. [16] studied the run of homozygosity (ROH) values together with population genetics statistics for the investigation of the genomic diversity in several heritage turkey breeds. Based on the 650K SNP genotyping data, at least 75% of birds had homozygous ROH regions, which are called ROH islands. The admixture analyses showed that six breeds, but not the Roma breed, were unique populations, indicating the conservation advantages of these heritage turkey breeds. Among the genes in the ROH islands, the PTGS2 and PLA2G4A genes on chromosome 10 were found to be related to reproduction efficiency, indicating the possible use of these genes for the improvement of the target trait(s).

The Japanese quail is a useful species in Asian countries, especially for the production of eggs. Shumaker et al. [17] performed genome sequencing for high stress (HS) and low stress (LS) lines of Japanese quail. Based on an SNP analysis of the two lines, they identified SNPs in relation to immune responses, DNA repair, and neurological signaling, which could possibly indicate stress factors affecting poultry production.

The last original paper regarding rare poultry species was about pigeons, and investigated the microRNA-associated-ceRNA networks for regulating crop milk production [18]. Based on the miRNAs expression profile of the female pigeon crop, 71 differentially expressed miRNAs were identified. Among them, miR-20b-5p, miR-146b-5p, miR-21-5p, and miR-26b-5p were found to be the key miRNAs regulating lactation. In the ceRNA network, miR-193-5p were located in the central position and three microRNAs were in the regulatory axes for lactation. This finding provides valuable information for the microRNA-associated-ceRNA networks with respect to crop milk production in pigeons.

Two review articles are published in this Special Issue. The first one concerns the molecular regulation of fat deposition in chicken by Nematbakhsh et al. [19]. This article includes several ways to decrease undesirable fat deposition using the selection of birds, feeding, including hormone supplementation, housing, and environmental changes. The paper also considers structural and functional regulatory mechanisms at the molecular level, including the regulatory mechanism of microRNAs (miRNAs) on lipid metabolism and fat deposition for the selection of birds with more desirable fat deposition. Finally, the second review paper by Park et al. [20] considers the use of cutting-edge technology for precise genome editing technologies mediated by the CRISPR/Cas9 system using cultured primordial germ cells (PGCs) and viral vector systems, with a particular focus on avian species and their application to agriculture and the biomedical industry.

The papers in this Special Issue cover various aspects of poultry genetics, breeding, and biotechnology related to chicken, goose, duck, turkey, Japanese quail, and pigeon. Furthermore, the studies in this Special Issue used a diverse range of methods from genomic technology to vaccine development, leading to state-of-the-art developments in this field of research, including perspectives on both current and future challenges regarding poultry species.
